# A Novel Linear Plasmid Mediates Flagellar Variation in *Salmonella* Typhi

**DOI:** 10.1371/journal.ppat.0030059

**Published:** 2007-05-11

**Authors:** Stephen Baker, Jonathan Hardy, Kenneth E Sanderson, Michael Quail, Ian Goodhead, Robert A Kingsley, Julian Parkhill, Bruce Stocker, Gordon Dougan

**Affiliations:** 1 The Wellcome Trust Sanger Institute, Hinxton, Cambridgeshire, United Kingdom; 2 Department of Pediatrics, Stanford University School of Medicine, Stanford, California, United States of America; 3 Department of Biological Sciences, University of Calgary, Calgary, Alberta, Canada; Max Planck Institute for Infection Biology, Germany

## Abstract

Unlike the majority of Salmonella enterica serovars, *Salmonella* Typhi (*S*. Typhi), the etiological agent of human typhoid, is monophasic. *S*. Typhi normally harbours only the phase 1 flagellin gene *(fliC),* which encodes the H:d antigen. However, some *S*. Typhi strains found in Indonesia express an additional flagellin antigen termed H:z66. Molecular analysis of H:z66+ *S*. Typhi revealed that the H:z66 flagellin structural gene *(fljB^z66^)* is encoded on a linear plasmid that we have named pBSSB1. The DNA sequence of pBSSB1 was determined to be just over 27 kbp, and was predicted to encode 33 coding sequences. To our knowledge, pBSSB1 is the first non-bacteriophage–related linear plasmid to be described in the Enterobacteriaceae.

## Author Summary

Flagella are whip-like structures found on the surface of bacterial cells that mediate swimming. Flagella contain a protein called flagellin, which is recognised as a danger signal by the immune system. *Salmonella* Typhi, the bacteria that causes typhoid fever, normally have flagella called H:d, but some strains only from Indonesia express distinct flagella, called H:z66. In this study we have located and sequenced the genes responsible for expressing these alternative flagella. Remarkably, these genes are located on a linear plasmid, an extra-chromosomal element that we have named pBSSB1. The significance of this finding is that linear plasmids are relatively common in bacterial species such as *Streptomyces* and *Borrelia*. However, such a linear element has never previously been described in enteric bacteria such as Escherichia coli and *Salmonella*. The identification of this novel linear plasmid in genetically tractable bacteria will facilitate future studies on the biology of linear plasmids and the pathogenicity of both flagella and *Salmonella* Typhi.

## Introduction

Flagella play a critical role in the lifestyle of many bacteria, and the flagellin subunit is an important target for pathogen recognition by the mammalian innate immune system through Toll-like receptor (TLR) 5 [1]. Antiserum against flagella (H antigen) and lipopolysaccharide (O antigen) are the cornerstone of *Salmonella* classification through the Kauffmann–White scheme [2], which divides Salmonella enterica into the various serovars. The majority of *S. enterica* serovars are biphasic, alternating expression between two flagellar antigens through a process called “phase variation” [3]. Only one of the two flagellin genes, *fliC* and *fljB,* which are located at distinct loci on the *Salmonella* chromosome, is expressed at any given time [4]. In contrast, S. enterica serovar Typhi (*S*. Typhi), the cause of the human systemic infection known as typhoid, is normally monophasic, harbouring only the phase 1 flagellin gene *fliC,* which encodes the H:d antigen, and lacking a *fljB* equivalent. However, some *S*. Typhi strains isolated in Indonesia express an alternative H antigen, known as H:j, and/or a second flagellin, called H:z66 [5]. Whilst H:j variants arise through a deletion within the *fliC* gene [6], the H:z66 antigen is encoded on an unlinked locus.

In 1981, Guinee et al. [7] described *S*. Typhi strains from Indonesia that were H:d and H:j negative but motile due to functional z66 flagella. Upon incubation with anti-z66 antiserum, these strains reverted to H:d or H:j, so the z66 antigen was presumed to be a phase 2 flagellum. Despite extensive screening of worldwide *S*. Typhi strain collections, the z66 antigen has only been detected in strains originating from Indonesia [5,8–10]. The z66 flagellin structural gene *(fljB^z66^)* (1,467 base pairs [bp]) has been previously isolated on a 3,325-bp DNA fragment cloned from a z66*+* strain of *S*. Typhi [11]. The same DNA fragment also encoded a putative phase 1 flagellin repressor *(fljA)* downstream of *fljB^z66^,* but the upstream region had no similarity to the site-specific inversion region of *hin,* which is associated with phase switching [12]. The genetic location of the gene encoding the z66 antigen *(fljB^z66^)* was not identified, and the DNA sequences beyond the original 3,325-bp cloned fragment have not previously been described. Here, we demonstrate that the *fljB^z66^* gene is located on a novel 27-kbp linear plasmid that has been isolated, sequenced, and shown to be capable of autonomous replication in Escherichia coli.

## Results

### The *fljB^z66^* Gene Is Located on a Plasmid

**Figure 1:**
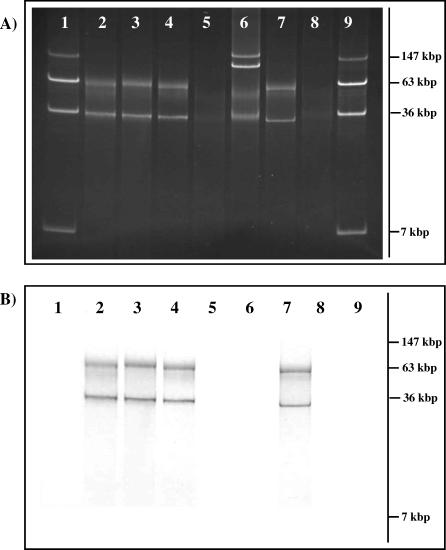
Identifying the Location of the H:z66 Flagellin Gene (A) Agarose gel electrophoresis of alkaline lysis preparation from H:z66+ and H:z66− strains. Lanes 1 and 9, E. coli 39R861; lane 2, *S*. Typhi 404Ty; lane 3, *S*. Typhi In20; lane 4, *S*. Typhi SGB32 (pBSSB2); lane 5, *S*. Typhi Ty2; lane 6, *S.* Typhi CT18; lane 7, E. coli SGB33 (pBSSB2), and lane 8, E. coli TOP10. (B) Southern blot of transferred DNA from (A). The transferred DNA was probed with a labelled PCR amplicon of the *fljB^z66^* gene and amplified using primers z66flag_F and z66flag_R (Table S2). Lanes are as shown in (A) and fragment sizes are estimated in comparison to (A).

*S*. Typhi strains isolated in Indonesia were tested for H:z66 antigen expression and for motility in soft agar. Two highly motile H:z66+ *S*. Typhi strains, In20 and 404Ty, were selected for further investigation. Despite DNA encoding *fljB^z66^* being readily cloned, it was not possible to map the gene onto the *S*. Typhi chromosome using Southern blotting following I-CeuI digestion (unpublished data). Subsequent analysis of DNA on agarose gels identified two candidate plasmids in *S*. Typhi In20 and 404Ty, one migrating at a similar speed to the 36-kbp circular plasmid marker, and another more diffuse band migrating with the 63-kbp marker (Figure 1A, lanes 2 and 3). DNA prepared from *S*. Typhi Ty2 yielded no plasmid DNA, whereas the multi-drug–resistant *S*. Typhi CT18 gave two plasmids corresponding to the previously characterised pHCM1 (218 kbp) and pHCM2 (106 kbp) [13,14]. To determine whether the novel candidate plasmids present in the z66*+ S*. Typhi strains encoded the *fljB^z66^* gene, a DNA fragment of the *fljB^z66^* gene was used to probe plasmid DNA preparations from the *S*. Typhi strains (Figure 1B). No hybridisation was detected with the DNA prepared from *S*. Typhi CT18 or Ty2 (Figure 1B, lanes 5 and 6). The z66+ strains, 404Ty (lane 2) and In20 (lane 3), yielded signals corresponding to both of the bands. The smaller band was identified as the native plasmid, named pBSSB1. The larger band may be an artefact of denaturation/renaturation during the alkaline lysis procedure because it is not visible with pulsed field gel electrophoresis (PFGE), which does not involve alkaline lysis. We suggest that it may be a complex renaturation product, rather than a simple linear molecule.

### Sequence Analysis of Plasmid pBSSB1

**Figure 2:**
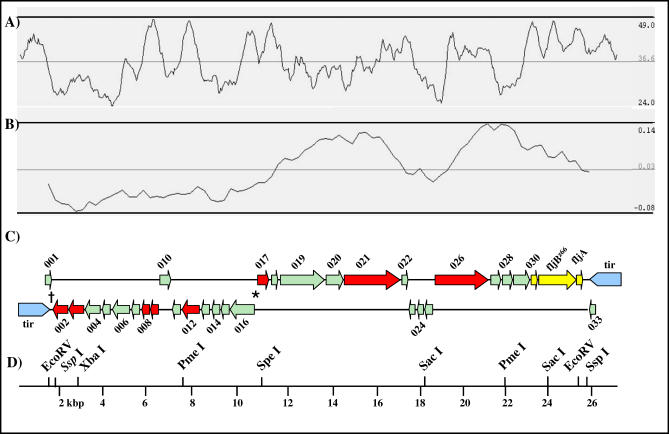
Map of the Complete Sequence of pBSSB1 (A) Plot of percentage GC content of pBSSB1, ranging from 24% to 49%, with an average of 36.6%. (B) Plot of GC skew ((G−C)/(G+C)), ranging from −0.08 to 0.14 and averaging 0.03. (C) CDS map of pBSSB1, consisting of the forward (upper) and the reverse (lower) DNA strand. The CDSs are identified with sequential numbers. The previously sequenced genes of Huang et al. [11] (030, *fljB^z66^*, and *fljA*) are labelled yellow. The tirs are labelled in blue, CDSs with similarity to previously described sequences are coloured red, and CDSs with no similarity to previous sequences are coloured green. *, the change in coding bias associated with a possible origin of replication. †, the location of the kanamycin resistance gene insertion. (D) Size scale in 2-kbp fragments of pBSSB1 (27,037 bp). Endonuclease cut sites are labelled for XbaI (2,708 bp), PmeI (7,704 and 21,995 bp), SacI (18,611 and 23,778 bp), and SpeI (11,137 bp); additionally, the cut sites located nearest the tirs are labelled for EcoRV (1,279 and 25,255 bp) and SspI (1,715 and 25,799 bp).

We tested the hypothesis that pBSSB1 was a conventional circular plasmid, but despite intensive cloning and sequencing efforts, were unable to generate a complete circular DNA sequence. Consequently, genomic DNA of *S*. Typhi 404Ty was sequenced by 454 Pyrosequencing, generating an 8-fold coverage of the entire genome. DNA sequence information derived from the plasmid was combined with sequences obtained by conventional cloning. Analysis of the subsequent linear plasmid sequence confirmed that it encoded the *fljB^z66^* gene and identified identical inverted repeat sequences present at both termini (terminal inverted repeats [tirs]) (Figures 2 and S1). Tirs are a common feature of linear plasmids in *Streptomyces* and *Borrelia* [15,16]. In *Streptomyces,* the size of the tirs varies from short palindromic repeats in SLP2 of S. lividans [17] to 95 kbp in plasmid pPZG101 of S. rimosus [18]. pBSSB1 has 1,230-bp tirs, with no similarity to other tirs and no direct, tandem, or palindromic repeats. The guanine-cytosine (GC) content of the tir regions, at 41%, is higher than the non-repetitive sequence. The linearity of the pBSSB1 sequence and the correct assembly of the tirs were confirmed by PCR (Figure S1).

pBSSB1 is 27,037 bp in length, which is 7 kbp less than the 34 kbp predicted by circular plasmid sizing on agarose gels (Figure 1A). Only the 3,456 bp of the region containing the *fljB^z66^* sequence [11] exhibited strong similarity to previously sequenced DNA in public databases. Annotation predicted 33 coding sequences (CDSs) (Figure 2; Table S1), only three of which (030, *fljB^z66^,* and *fljA*) have been previously described [11]. The predicted coding density of the plasmid is one gene per 1.257 kbp (85.4%); this is similar to the chromosome and plasmids pHCM1 and pHMC2 from *S*. Typhi CT18 (87.6%, 83.8%, and 87.1%, respectively) [19]. The GC content of pBSSB1 is 36.6% (Figure 2). This is substantially lower than the GC content of genomes of enteric bacteria (∼50%–52%), suggesting that the plasmid may originate from a non-enteric source. Additionally, the sequence is not significantly similar to any previously described plasmids or bacteriophage. At the least stringent BLAST E-value applicable (<0.01), the sequence demonstrates no similarity to any described bacteriophage proteins. Moreover, of the 30 novel coding regions, 22 do not demonstrate any similarity to any previously described DNA or amino acid sequence in public databases. The 22 CDSs with no similarity to previously sequenced DNA are coloured green in Figure 2. The previously sequenced region includes the gene encoding the putative *fliC* repressor *(fljA),* the gene encoding the z66 flagellin antigen *(fljB^z66^),* and a gene of unknown function (030) immediately upstream of *fljB^z66^*. This region in pBSSB1 is 99% identical to the sequence produced by Huang et al. [11]. 030*, fljB^z66^,* and *fljA* are the final three CDS on the forward strand, adjacent to the inverted repeat (coloured yellow in Figure 2), with a combined GC content of 43%, implying that they may be a more recent acquisition by the element. Further analyses and annotation of the CDSs encoded on pBSSB1 are included in Table S1.

A change in GC skew ((G−C)/(G+C)) suggests that the region immediately upstream of 017 may act as a bi-directional origin of replication for pBSSB1 (distinguished by an asterisk in Figure 2). Changes in GC skew are often associated with the origin of replication on plasmids and bacterial chromosomes [20] and have been used previously to predict the internal origin of bi-directional linear replication [21]. The direction of transcription of the majority of genes is consistent with this. It is also known that linear *Streptomyces* plasmids with tirs, including pSLA2, replicate divergently from a central origin towards the termini [22]. No short DNA repeats, commonly associated with replication origins, were found within this region in pBSSB1. However, CDS 017 contains an ATP-binding motif similar to those found in partition proteins from some plasmids, but otherwise has no overall similarity to these proteins.

### Transformation of E. coli with pBSSB2

A kanamycin resistance marker was inserted within pBSSB1 at position 1,295 bp (indicated by a dagger in Figure 2; Figure S1) to facilitate experimental analysis. The plasmid with the kanamycin resistance cassette was named pBSSB2 and the modified *S*. Typhi In20 was named SGB32. Plasmid pBSSB2 was isolated from SGB32 and yielded a plasmid pattern indistinguishable from that of *S*. Typhi 404Ty and *S*. Typhi In20, despite the insertion of the 1,432-bp cassette (Figure 1A, lane 4). E. coli TOP10 cells were electrotransformed with purified pBSSB2 DNA isolated from *S*. Typhi SGB32, and kanamycin-resistant colonies were obtained. One kanamycin-resistant transformant was designated E. coli SGB33. Plasmid DNA from E. coli SGB33 (Figure 1A and 1B, lane 7) was indistinguishable from that of the *S*. Typhi strains harbouring pBSSB1, and subsequent Southern blotting confirmed the presence of the *fljB^z66^* gene. pBSSB2 was stably inherited by *E. coli* SGB33, even in the absence of antibiotic selection. Expression of the z66 antigen could not be detected in E. coli SGB33 using Western blotting (unpublished data). We hypothesised that undetectable z66 antigen expression in E. coli was due to differences in flagellar regulation between the different bacterial species. A z66− *S*. Typhi strain was transformed with pBSSB2 DNA isolated from E. coli SGB33, the plasmid was stably maintained, and the z66 antigen was dominantly expressed.

### Molecular Analysis of pBSSB1

**Figure 3:**
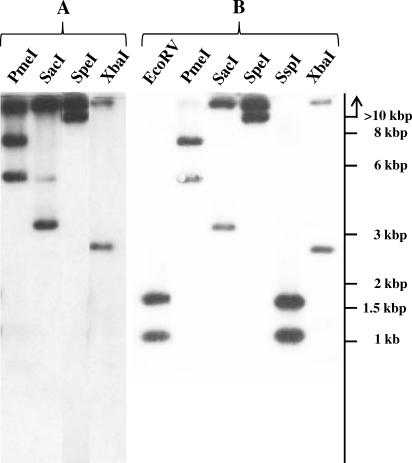
Southern Blotting Analysis of pBSSB1 (A) Southern blot of transferred digested genomic DNA from *S*. Typhi 404Ty, probed with purified and labelled pBSSB1 plasmid DNA. Sizes are estimated with respect to Hyperladder I, as measured prior to transfer. Predicted digestion sites from the DNA sequence are highlighted in Figure 2. (B) Southern blot of the tir region of pBSSB1 using labelled DNA prepared from a PCR amplicon from *S*. Typhi 404Ty DNA (primers tir_F and tir_G, Table S2) as a probe against digested genomic DNA from *S*. Typhi 404Ty.

**Figure 4:**
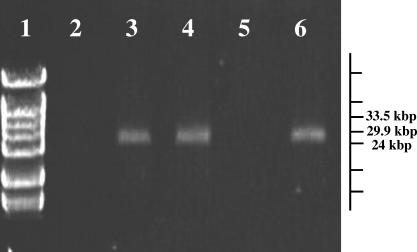
Molecular Analysis of pBSSB2 PFGE of agarose plugs prepared with E. coli SGB33 or pBSSB2 DNA. Lane 1, Hyperladder VI; lane 2, E. coli TOP10; lane 3, untreated E. coli SGB33; lane 4, pBSSB2 DNA treated with S1 nuclease; lane 5, pBSSB2 DNA treated with exonuclease III; lane 6, pBSSB2 DNA treated with lambda exonuclease.

The linearity of pBSSB1 was confirmed experimentally by probing *S.* Typhi 404Ty genomic DNA cleaved with PmeI, SacI, SpeI, and XbaI with pBSSB1 (Figure 3A). Restriction endonucleases that were predicted to cut once (SpeI and XbaI) generated two DNA fragments, and those predicted to cut twice (PmeI and SacI) generated three DNA fragments (Figure 3A). The size of the observed restriction fragments corresponds to the sizes for linear pBSSB1 DNA predicted by the *in silico* digestion described in Figure 2. pBSSB2 DNA from E. coli SGB33 was also embedded in agarose plugs and incubated with S1 nuclease, which linearizes supercoiled circular DNA [23,24]. S1 nuclease activity was proven using purified pUC18 DNA. S1 nuclease failed to alter the mobility of pBSSB2 after incubation for 1 h (Figure 4, lane 4), consistent with linearity of the element. pBSSB2 DNA was entirely degraded by 3′-5′ exonuclease III (Figure 4, lane 5) but not by lambda exonuclease, which digests in a 5′-3′ direction (Figure 4, lane 6). Activity of both exonucleases was demonstrated on linearized pUC18 DNA. Additionally, omitting the proteinase from the preparation of plugs for PFGE prevented pBSSB2 mobilisation into the agarose gel (unpublished data). These data suggest that pBSSB1 may be similar to linear plasmids from *Streptomyces* in having protein covalently bound to the 5′ end of the DNA and not palindromic hairpin loops at the telomeres as found in other enteric linear elements, such as bacteriophage N15 from E. coli [25].

Cleaved genomic DNA from 404Ty was probed with a PCR amplicon of the tir sequence to determine if pBSSB1 was additionally inserted into the chromosome. If pBSSB1 is solely in an extra-chromosomal form, only two fragments corresponding to the tir would be detected. However, if pBSSB1 was also inserted in the chromosome, further DNA fragments would be expected. Figure 3B shows the Southern blotting analysis using the tir-generated probe against genomic 404Ty DNA cleaved with different restriction endonucleases. Only the two DNA fragments predicted to originate from linear pBSSB1 were detected, suggesting that the plasmid was not inserted into the chromosome at a detectable level.

## Discussion

Linear DNA replicons are extremely rare in enteric bacteria, and those that have been described, including PY54 of Yersinia enterocolitica [26], N15 of E. coli [25], and PKO2 of Klebsiella oxytoca [27], are linear hairpin-ended prophage. To our knowledge, pBSSB1 is the first linear element to be described in the Enterobacteriaceae that bears no detectable sequence homology to bacteriophage. The fact that a linear plasmid can exist and replicate in a pathogenic member of the Enterobacteriaceae and have an impact on the phenotype of the bacteria is a significant observation. The identification of pBSSB1 will facilitate future studies on the biology of such linear extra-chromosomal elements in other bacterial species. Despite pBSSB1 having no sequences common to previously described elements, it does share some structural features of known linear plasmids*.* Unlike other linear elements in enteric bacteria, pBSSB1 contains tirs, and our data demonstrate that the ends are capped with covalently bound protein, as found in *Streptomyces* linear plasmids [28,29], and not closed hairpin loops. The tir, GC skew, and coding bias suggest that pBSSB1 replicates from a central internal origin, as do all small and large *Streptomyces* linear plasmids, such as pSLA2 from S. rochei [28,29].

Global analysis of *S.* Typhi isolates suggests that genome variation is extremely limited in this pathogen [30,31]. Indeed, plasmids are relatively rare in this serovar and are generally restricted to members of the IncH1 family [14]. How and why *S*. Typhi acquired this element is open to speculation. Flagella play a critical role in the lifestyle of bacteria and are an important target of pathogen recognition by the mammalian innate immune system via the TLR5 pathway [1,32]. The incidence of typhoid in Indonesia is one of the highest in the world [33,34], and the fact that circulating *S.* Typhi strains have acquired and maintained an additional flagellin gene may be related to the population dynamics of typhoid infections in the region. H:z66+ strains have been identified only in this location, and although the H:d variant, H:j, has been isolated elsewhere, it is highly prevalent in Indonesia [35]. It is possible that there is significant immune selection ongoing within this *S*. Typhi population. This may be expected for a pathogen that causes systemic infection and has the potential to exist in a persistent state. Currently, we do not know if the presence of the z66 flagella or pBSSB1 impacts the pathogenicity of *S*. Typhi strains, nor is it readily possible to test this hypothesis in the laboratory, because *S*. Typhi only causes disease in humans.

The influence of gene gain via horizontal transfer on the pathogenesis of various *Salmonella* serovars has been well documented. The acquisition of novel DNA sequences by *S*. Typhi in Indonesia may have allowed them to adapt to a new niche, or may have increased their fitness within their prior niche.

## Materials and Methods

### Bacterial strains.

*S.* Typhi In20 (H:z66+) and *S*. Typhi 404Ty (H:z66+) were isolated in Indonesia and were provided by Leon LeMinor (*Salmonella* Genetic Stock Centre, Calgary, Alberta, Canada). *S*. Typhi CT18 (H:z66−) and *S*. Typhi Ty2 (H:z66−), for which complete genome sequences exist, are from The Sanger Institute strain collection. *S*. Typhi In20 was transformed with pKD46 (*S*. Typhi SGB31) and the resulting kanamycin-resistant strain was named *S*. Typhi SGB32 (this study). High efficiency E. coli TOP10 (Invitrogen, http://www.invitrogen.com) were used to demonstrate the transferable nature of pBSSB2. Transformed E. coli TOP10 containing pBSSB2 was named E. coli SGB33 (this study). Transformed *S.* Typhi BRD948 containing pBSSB2 and expressing H:z66 was named *S*. Typhi SGB34 (this study). E. coli 39R861 was used for sizing plasmid extractions on agarose gels and contains plasmids of 7, 36, 63, and 147 kbp.

### Plasmid isolation.

Plasmid DNA was prepared using an alkaline lysis method originally described by Kado and Liu [36]. The resulting plasmid DNA was separated by electrophoresis in 0.7% agarose gels made with 1x E buffer. Gels were run at 90 V for 3 h, stained with ethidium bromide, and photographed. High purity plasmid DNA was isolated for transformation using alkaline lysis and either AgarACE purification (Promega, http://www.promega.com) or ultra-centrifugation based upon a method described by Taghavi et al. [37].

### Southern blotting.

Southern blotting was carried out using Hybond N+ nitrocellulose. Probes were prepared from purified PCR products (PCR purification kit; Qiagen, http://www.qiagen.com) amplified using primers outlined in Table S2, or from purified pBSSB1 DNA. Purified PCR products or plasmid DNA was labelled using the Gene Images CDP-Star and AlkPhos Direct Labeling kit (GE Healthcare, http://www.gehealthcare.com). Detection was performed with the Gene Images CDP-Star Detection kit. The sizes of restriction fragments were estimated by comparing migration distances against Hyperladder I (Bioline, http://www.bioline.com).

### Insertion of kanamycin cassette.

A kanamycin resistance gene was inserted into pBSSB1 using a modified version of the lambda red recombinase (one-step method) described by Datsenko and Wanner [38]. PCR products were amplified in ten 50-μl reactions with the primers described in Table S2 using pKD4 DNA as a template. PCR-amplified DNA was pooled, precipitated, and re-suspended in 10 μl of nuclease-free water. Re-suspended DNA was mixed with 50 μl of competent *S*. Typhi SGB31 cells (grown in LB broth, supplemented with 0.1 M arabinose, and harvested at 0.3 OD_600_) in 2-mm electroporation cuvettes (Invitrogen). Cells were electrotransformed (2.4 kV, 600 ohms, 25 μF; Bio-Rad Gene Pulser, http://www.bio-rad.com), allowed to recover for 2 h statically at 37 °C in 400 μl of SOC, and then plated onto LB medium supplemented with 25 μg/ml kanamycin.

### DNA cloning, sequencing, and annotation.

An H:z66 cosmid was constructed by cloning the *fljB^z66^* region into the BamHI site of vector cosmid p14B1 using a partial Sau3A digestion. The insert was shotgun sub-cloned into pUC18, sequenced, and annotated as previously described [39]. The sequence of pBSSB1 was completed by supplementing the cosmid insert sequence with draft data of *S*. Typhi 404Ty produced by 454 Pyrosequencing (454 Life Sciences, http://www.454.com) [40].

### Molecular analysis.

The linear nature of pBSSB2 was demonstrated using PFGE (CHEF DRII, Bio-Rad). Agarose plugs containing lysed bacterial cells were prepared using the CHEF Bacterial Genomic DNA Plug Kit (Bio-Rad) as recommended by the manufacturer. 1.2% agarose gels (0.5x TBE) were loaded with the genomic DNA plugs, and samples were electrophoresed for 16 h at 6 V/cm, 14 °C, 1–6 seconds switch time, in 0.5x TBE and stained with ethidium bromide. Band sizing was estimated by comparison to the migration of Hyperladder VI (Bioline). S1 nuclease, exonuclease III, and lambda exonuclease treatment was performed on pBSSB2 DNA in agarose plugs as previously described [24]. Activity of the enzymes was confirmed on linear and circular pUC18 DNA.

## Supporting Information

Figure S1 The tirs of pBSSB1 (A) Map of the terminal (3 kbp) at either end of pBSSB1 showing the tirs and the adjacent genes. The locations of the primer sites for the lambda red recombinase kanamycin insertion are highlighted by pink arrows marked 1, 2, 3, and 4; these correspond to primers z66_red_1 to z66_red_4, respectively (Table S1). The locations of the primers for PCR probing of the tirs are shown by yellow arrows a, b, c, d, and e, which correspond to primers tir_a to tir_e, respectively (Table S1).
(B) Agarose gel of PCR amplicons produced within the tirs. Upper bands were amplified using DNA from *S*. Typhi In20 as a template; lower bands were amplified using DNA from E. coli SGB33 as a template. Sizes are estimated in comparison to Hyperladder I (Hp). Lanes correspond to the combination of primers used in the PCR reaction; these are designated in (A). Amplicons produced by a+d and b+d using DNA from E. coli SGB33 as the template are approximately 1.5 kbp larger than those of *S*. Typhi In20; this is due to the kanamycin cassette insertion at position 1,295.
(192 KB PPT)

Table S1 The Annotation of pBSSB1 Table S1 includes the sequential CDS numbers, the strand of the CDSs, the location of the CDSs, and the size (amino acids) of the putative protein encoded by the CDSs. Any features or hypothetical function of the putative proteins are also given. For putative proteins with significant BLASTP hits against the GenBank database, the organism, locus tag designation, and E-value of the best hit is provided.
(60 KB XLS)

Table S2 Names, Amplification Targets, and Sequences of the Primers Used in This Study (14 KB XLS)

### Accession Numbers

The pBSSB1 sequence and annotation is available from Genbank/EMBL (http://www.ncbi.nlm.nih.gov/Genbank) under accession number AM419040. The GenBank accession numbers for other sequences discussed in the manuscript are *fljB^z66^* gene (AB108532), *S*. Typhi CT18 (NC_003198), and *S*. Typhi Ty2 (NC_004631).

## Abbreviations

bp: base pair
CDS: coding sequence
GC: guanine-cytosine
PFGE: pulsed field gel electrophoresis
*S*. Typhi: *Salmonella enterica* serovar Typhi
tir: terminal inverted repeat
