# Supplementary material for: A Novel Linear Plasmid Mediates Flagellar Variation in Salmonella Typhi
Source: PLoS Pathog. 2007 May 11;3(5):e59. doi: 10.1371/journal.ppat.0030059 (PMC1876496; doi:10.1371/journal.ppat.0030059)
Supplement: Figure S1 — (A) Map of the terminal (3 kbp) at either end of pBSSB1 showing the tirs and the adjacent genes. The locations of the primer sites for the lambda red recombinase kanamycin insertion are highlighted by pink arrows marked 1, 2, 3, and 4; these correspond to primers z66_red_1 to z66_red_4, respectively (Table S1). The locations of the primers for PCR probing of the tirs are shown by yellow arrows a, b, c, d, and e, which correspond to primers tir_a to tir_e, respectively (Table S1). (B) Agarose gel of PCR amplicons produced within the tirs. Upper bands were amplified using DNA from S. Typhi In20 as a template; lower bands were amplified using DNA from E. coli SGB33 as a template. Sizes are estimated in comparison to Hyperladder I (Hp). Lanes correspond to the combination of primers used in the PCR reaction; these are designated in (A). Amplicons produced by a+d and b+d using DNA from E. coli SGB33 as the template are approximately 1.5 kbp larger than those of S. Typhi In20; this is due to the kanamycin cassette insertion at position 1,295. (192 KB PPT) [file ppat.0030059.sg001.ppt]

## Slide 1
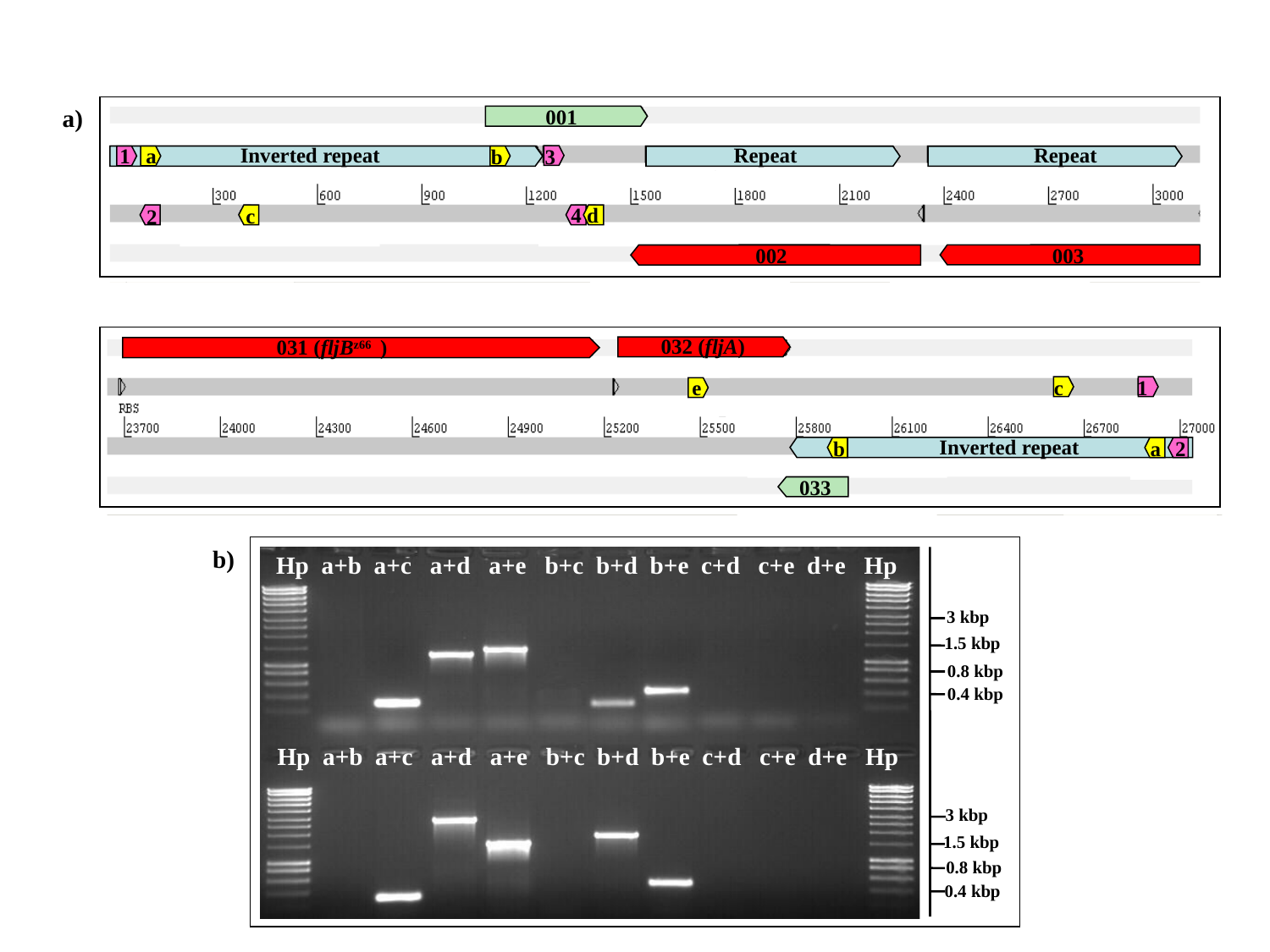

a)
001
Inverted repeat
Repeat
Repeat
 1 a
 b 3
Repeat
 4 d
 c
2
003
002
5
031 (fljBz66 )
032 (fljA)
 c 1
 e
Inverted repeat
 b
 a
 2
TIR
 A 2
033
b)
Hp a+b a+c a+d a+e b+c b+d b+e c+d c+e d+e Hp
 3 kbp
1.5 kbp
0.8 kbp
0.4 kbp
Hp a+b a+c a+d a+e b+c b+d b+e c+d c+e d+e Hp
 3 kbp
1.5 kbp
0.8 kbp
0.4 kbp
